# Risk of Presenting with Poor-Prognosis Metastatic Cancer in Adolescents and Young Adults: A Population-Based Study

**DOI:** 10.3390/cancers14194932

**Published:** 2022-10-08

**Authors:** Jessica K. Sheth Bhutada, Amie E. Hwang, Lihua Liu, Kai-Ya Tsai, Dennis Deapen, David R. Freyer

**Affiliations:** 1 Cancer and Blood Disease Institute, Children’s Hospital Los Angeles, Los Angeles, CA 90027, USA; 2 Department of Pediatrics, Keck School of Medicine, University of Southern California, Los Angeles, CA 90033, USA; 3 USC Norris Comprehensive Cancer Center, Los Angeles, CA 90033, USA; 4 Los Angeles Cancer Surveillance Program, Los Angeles, CA 90089, USA; 5 Department of Population and Public Health Sciences, Keck School of Medicine, University of Southern California, Los Angeles, CA 90089, USA; 6 Department of Medicine, Keck School of Medicine, University of Southern California, Los Angeles, CA 90033, USA

**Keywords:** Adolescents, young adults, incidence, race/ethnicity, sex, socioeconomic status, metastatic cancer, metastatic disease, AYAs

## Abstract

**Simple Summary:**

Despite significant survival improvements for adolescents and young adults (AYAs) with cancer overall, several subsets of AYAs lag behind. This is especially true for AYAs diagnosed with metastatic disease, where, for many types of cancer, fewer than 20% of patients are alive 5 years later. Which sociodemographic subgroups of AYAs are at highest risk for presenting with metastatic disease is not well understood. In this study using United States Surveillance, Epidemiology, and End Results Program data, we found significantly increased risk of metastatic disease across many cancer sites for non-Hispanic Blacks, males, and low socioeconomic status AYAs. In particular, metastatic melanoma was striking for demonstrating multiple sociodemographic disparities. Results of this study shed light on the relative roles of age, race/ethnicity, sex, and socioeconomic status in metastatic disease presentation. These will inform future, cancer-specific research to elucidate biological, social, and environmental mechanisms underlying these associations with metastatic disease.

**Abstract:**

Having metastatic disease at diagnosis poses the great risk of death among AYAs with cancer from all sociodemographic subgroups. This “landscape” study utilized United States Surveillance, Epidemiology, and End Results Program data from 2000–2016 to identify subgroups of AYAs at highest risk for presenting with metastases across twelve cancer sites having a poor-prognosis (5-year survival <50% with metastases). Adjusted odds ratios for risk of metastatic disease presentation were compared for AYAs in aggregate and by sociodemographic subgroup (race/ethnicity, sex, socioeconomic status [SES]). In general, AYAs who were male, racial/ethnic minorities, or low SES were at consistently greatest risk of metastases. Strikingly, having metastatic melanoma was independently associated with multiple AYA sociodemographic subgroups, including males (aOR 3.11 [95% CI 2.64–3.66]), non-Hispanic Blacks (4.04 [2.32–7.04]), Asian Pacific Islanders (2.99 [1.75–5.12]), Hispanics (2.37 [1.85–3.04]), and low SES (2.30 [1.89–2.80]). Non-Hispanic Blacks were more likely to present with metastatic cancer in all sites, except for bone, rhabdomyosarcoma, and stomach. Low SES AYAs are more likely to present with metastatic melanoma, bone tumors, soft tissue sarcomas, breast, cervical, lung, and stomach carcinomas. Building on these results, future cancer-specific studies should investigate the connection between sociodemographic risk factors and biological drivers of metastases. This line of research has potential to inform targeted public health and screening efforts to facilitate risk reduction and earlier detection of these deadly diseases.

## 1. Introduction

Although 5-year aggregate survival for newly diagnosed cancer in adolescents and young adults (AYAs, 15–39 years old) now exceeds 85%, it is poorer for certain patient subsets, including racial/ethnic minorities, males, and those of low socioeconomic status (SES) [1,2,3,4]. However, being diagnosed with metastatic disease carries the worst prognosis by far. In AYAs with metastatic cancer, five-year survival is below 40% for most types and much lower for many. For metastatic breast and colorectal carcinoma, 5-year survival is only 15–20% and is less than 10% for metastatic melanoma and metastatic carcinomas of the kidney, stomach, and lung [5,6]. In aggregate, adjusted mortality for AYAs diagnosed with metastatic cancer is 6-fold greater than those with localized disease [1]. For breast, lung, stomach, and colorectal carcinoma, as well as soft tissue sarcomas, AYAs with metastatic versus localized disease have an 8 to 14-fold higher risk of death, and a 30-fold higher risk with metastatic melanomas and carcinomas of the uterus and kidney [1]. These risks equate to substantial numbers of patients, as exemplified by newly diagnosed metastatic breast and colon cancer, which affect approximately 880 and 1296 AYAs annually [2].

Factors driving risk of metastatic disease presentation in AYAs are much less clear. It has been postulated that AYAs, compared to younger and older patients, are generally more prone to developing high-risk cancers due to a propensity for adverse tumor biology and clinically aggressive disease, delayed diagnosis, and limited access to care [6,7,8,9,10,11]. Aggressive tumor biology is associated with higher risk of metastases in certain malignancies such as alveolar rhabdomyosarcoma, which is more likely to present in AYAs than children [12]. Underlying mechanisms driving aggressive tumor biology remain unknown for most cancer sites but genetic ancestry, i.e., inheritance of higher risk alleles within racial/ethnic subgroups, has been implicated in diseases such as HER2/ER/PR-negative (“triple-negative”) breast cancer [13,14]. Additionally, limited studies suggest that increased allostatic load as a measure of toxic stress represents a plausible mechanism for the development of aggressive breast cancer in young Black women [15]. There may also be underlying differences in sex-specific hormones that mediate the increased risk of mortality in younger men compared to women for cancers such as colorectal carcinoma [16,17]. In older adults, while several environmental and behavioral risk factors such as smoking, obesity, diet, carcinogens, and infection have been implicated in the development of certain AYA-relevant cancers such as lung, stomach, and kidney carcinomas, there is no established association of these factors with increased risk of metastatic disease [18,19,20,21,22]. Those risk factors often accompany lower SES, lower education, lower health literacy, limited green space, limited access to healthy foods, and tobacco sales [23,24,25,26,27,28].

Delays in diagnosis could also contribute to presentation with metastatic disease in AYAs. This could result from limited access to diagnostic and therapeutic care due to insurance barriers, and from limited access to specialized treatment facilities, factors that may be associated with socioeconomic status [10,29,30]. Additionally, low provider and patient awareness of the possibility of cancer in AYAs, coupled with systematic barriers to healthcare access, may contribute to delayed care for “red flag symptoms,” and lead to multiple provider referrals prior to reaching a definitive diagnosis [31]. Cervical cancer is the only cancer site with defined screening guidelines for AYAs. Despite many efforts to improve screening for racial/ethnic minority and low socioeconomic status AYAs, these subgroups of AYAs remain under screened, potentially contributing to their high rates of metastatic cervical cancer [32]. In response to the rising incidence of young-onset colorectal cancer, the minimum age for general screening has been lowered from 50 to 45 years of age [33]. Although there are currently no screening guidelines for melanoma recommended by the United States Preventive Task Force (USPSTF), this topic is actively being re-evaluated [33]. By identifying and understanding the characteristics of AYA populations at greatest risk of metastatic disease, more focused screening efforts for vulnerable groups of AYAs can be prioritized.

Given these concerns, it is somewhat surprising that patterns of metastatic disease among AYAs and their potential relationship with sociodemographic risk factors remain largely unexplored. Associations between sociodemographic and biological variables are documented for some high-risk metastatic and loco-regional cancers, including triple-negative breast cancer in younger Black women, colorectal cancer in younger patients and racial/ethnic minorities, and EGFR-mutant lung cancer in young Asians [34,35,36,37,38,39,40,41,42,43,44]. AYAs who are racial/ethnic minorities or of low SES have a significantly higher risk of presenting with metastatic disease in breast, stomach, and kidney cancer than older adults [45]. However, within the AYA population, the extent to which specific sociodemographic subgroups may be at increased risk for presenting with metastatic versus localized cancer is not well-studied. Such information could shed light on the relative impact of social/environmental risk factors versus aggressive tumor biology contributing to late-stage diagnosis in this vulnerable population.

Therefore, we utilized data from the Surveillance, Epidemiology, and End Results (SEER) registry to compare sociodemographic risk factors for AYAs presenting with metastatic versus loco-regional disease. To help identify patterns informative about AYAs as a whole, we utilized a “landscape” approach and selectively evaluated twelve poor-prognosis cancers whose 5-year survival with metastatic disease is less than 50%. We hypothesized that specific sociodemographic subgroups would show consistently increased risk of metastases across multiple cancer sites, thus indicating broader cancer care inequities, such as impaired access and delayed diagnosis.

## 2. Materials and Methods

This was a population-based retrospective cohort study utilizing SEER-18 registry data. Patients were 15–39 years old when first diagnosed with one of a group of poor-prognosis, metastatic primary malignancies (pragmatically defined as common solid tumors relevant to AYAs with 5-year overall survival less than 50%) between January 2000 and December 2016. Stage of disease was classified as loco-regional versus metastatic (“distant”) disease, defined by the SEER coding rule as “tumor which has spread to body areas distant or remote from the primary tumor” [46]. Cancer sites included in this analysis were bone tumors (osteosarcoma, chondrosarcoma, Ewing sarcoma, and others), melanoma, rhabdomyosarcoma, other soft tissue sarcomas, and carcinomas of the breast, cervix, uterus, ovary, colon-rectum, kidney, lung, and stomach. Rhabdomyosarcoma was evaluated separately as it is clinically and biologically distinct from other soft tissue sarcomas [47]. Cervical and uterine cancers were examined individually due to differences in biology, risk factors, and screening. Consistent with our focus on prevalent poor-prognosis cancers, germ cell tumors, Hodgkin lymphoma, and thyroid cancers were excluded as their 5-year survival is > 50% even with metastases [5]. Kaposi sarcoma and non-Hodgkin lymphoma were excluded due to their distinctive HIV-associated epidemiology [1,48]. Leukemias were excluded as they are inherently disseminated and not staged as metastatic or non-metastatic. Patients with subsequent primary cancers or unknown disease stage were excluded.

### 2.1. Variable Definitions

For each case, age, sex (male, female); race and ethnicity (non-Hispanic White [NHW], non-Hispanic Black [NHB], non-Hispanic Asian/Pacific Islander [NHAPI], and Hispanic [all races]); and SES were assessed. The SEER census tract level SES index is a time-dependent composite score constructed from seven relevant census tract variables: median household income, median house value, median rent, percent below 150% of poverty line, education index, percent working class, and percent unemployed [49,50]). SES scores are calculated for each year using census data and American Community Survey 5-year estimates and subsequently categorized into tertiles with equal populations across the entire SEER catchment area. Tertiles were chosen instead of quintiles to optimize case numbers for all cancer types and were accessed through the SEER specialized census-tract level and rurality database.

### 2.2. Statistical Analyses

Incidence data were obtained using SEER*Stat software version 8.3.6 [51]. The primary outcome was metastatic disease presentation. Proportions of metastatic disease were calculated and compared using chi-square analysis for AYAs in aggregate, by cancer site, and by sociodemographic subgroup. Localized and regional disease were combined to better highlight the different proportions of metastatic disease. The differences in proportions of the subgroups were compared for each sociodemographic variables using chi-square analysis. Univariate and multivariate logistic regression analysis was performed to determine the relative risk of metastatic disease presentation for AYAs in aggregate and by cancer site for younger patients (age 15–29 years), males, NHBs, NHAPIs, Hispanics, and low and middle SES (reference groups: older patients [age 30–39 years], females, NHWs, and high SES, respectively). Multivariate models were adjusted for all covariates (age, sex, race/ethnicity, and SES) to estimate adjusted odds ratios (aORs) with 95% confidence intervals (95% CI). All *p*-values were two-sided with significance defined as *p* < 0.05. All statistical analyses were performed using SEER*Stat Version 8.3.6 and SAS Version 9.4.

## 3. Results

### 3.1. Stage Distribution

**Figure 1:**
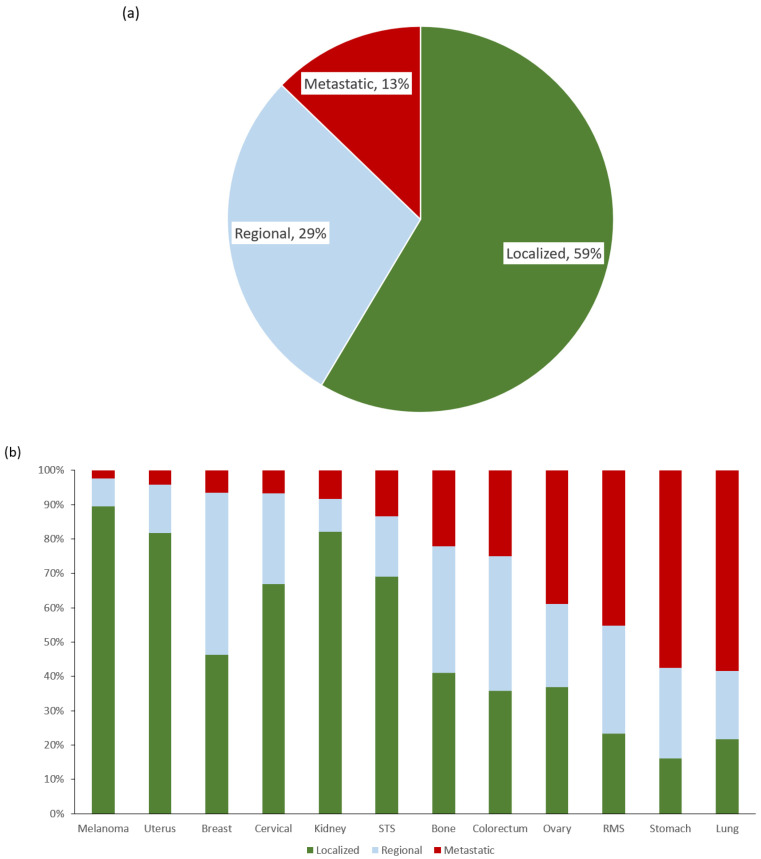
(**a**) Stage distribution for poor-prognosis metastatic cancers in aggregate. (**b**) Proportion of metastatic disease by cancer site. Total percentage in Figure 1A is greater than 100 due to rounding to nearest whole number.

For all these poor-prognosis sites combined, the overall proportion of AYAs having metastatic disease was 13% (Figure 1a). The proportion with metastatic disease varied significantly by cancer site, ranging from 2% in melanoma to 56% in lung carcinoma (Figure 1b). Stomach and lung were the only two sites where AYAs were more likely to present with metastatic than loco-regional disease (53.7% and 56.5%, respectively, Figure 1b). The overall proportion of AYAs with metastatic disease was statistically significantly different, yet clinically similar among those who were younger (15–29 years) versus older (13% vs. 12%, *p* = 0.001), but significantly higher for males versus females (19% vs. 10%, *p* < 0.001), racial/ethnic minorities versus NHWs (16–18% vs. 10%, *p* < 0.001 for all), and lower versus higher SES (16% vs. 10%, *p* < 0.001, Table 1).

### 3.2. Risk of Metastatic Disease for Sociodemographic Subgroups

**Figure 2:**
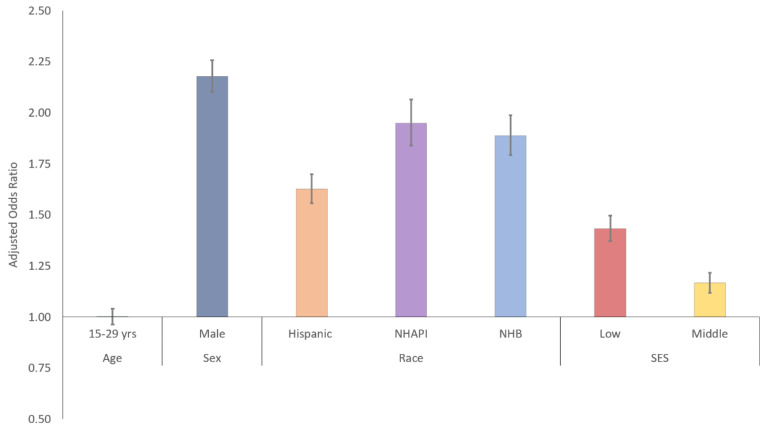
Risk of metastatic disease by sociodemographic subgroup for poor-prognosis cancers in aggregate.

For all cancers in aggregate, there was no overall difference in the likelihood of AYAs having metastatic disease based on age (aOR 1.00, 95% CI 0.96–1.04, Figure 2, Table 2). Male AYAs were at higher risk of metastases compared to females (aOR 2.18, 95% CI 2.11–2.26). Minorities were at overall higher risk of metastatic disease; NHAPIs and NHBs were at highest risk (aOR 1.95 [95% CI 1.82–2.04] and 1.89 [95% CI 1.77–1.96], respectively). Low and middle SES patients were also at a higher risk of metastatic disease presentation (aOR 1.43 [95% CI 1.36–1.48] and 1.17 [95% CI 1.11–1.21], respectively).

#### 3.2.1. Age

**Figure 3:**
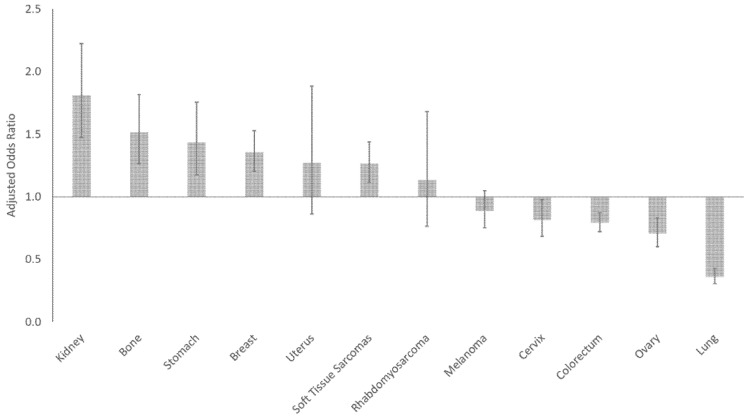
Risk of metastatic disease for younger AYAs by cancer site. Reference group: older AYAs (30–39 years).

After adjusting for race/ethnicity, sex, and SES, younger AYAs were statistically significantly more likely to present with metastatic kidney, bone, stomach, breast cancer and soft tissue sarcomas (Figure 3, Table 2). In contrast, metastatic cervical, colorectal, ovarian and lung cancer were more likely to occur in older AYAs.

#### 3.2.2. Sex

**Figure 4:**
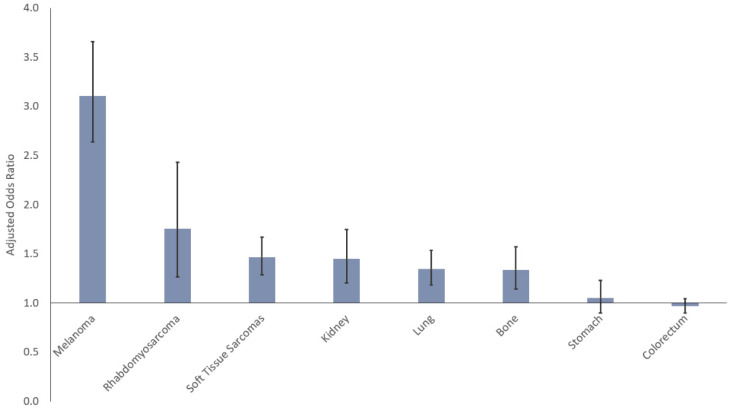
Risk of metastatic disease for male AYAs by cancer site. Reference group: female AYAs.

After adjusting for all other sociodemographic factors, the risk for presenting with metastatic disease was higher for male than female AYAs for all cancers except colorectal and stomach cancers (Figure 4, Table 2). The risk for males presenting with metastases was greatest in melanoma (aOR 3.11 [95% CI 2.64–3.66]).

#### 3.2.3. Race/Ethnicity

**Figure 5:**
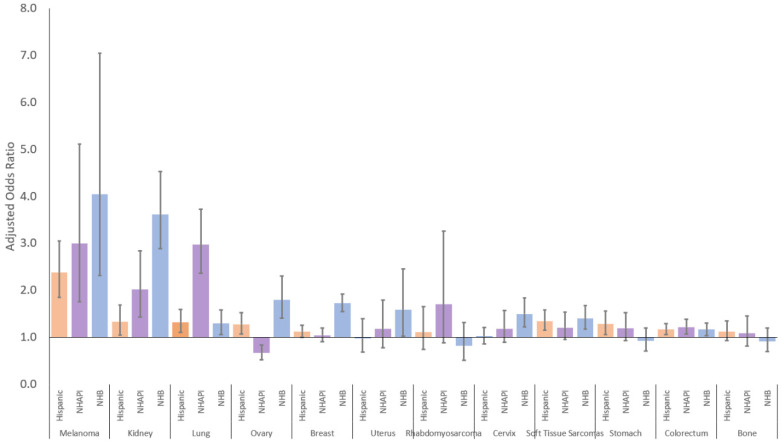
Risk of metastatic disease for racial/ethnic minority AYAs by cancer site. Reference group: non-Hispanic Whites.

After adjusting for other sociodemographic variables, racial/ethnic minorities were at higher risk of metastatic disease compared to NHWs in most cancer sites (Figure 5, Table 2). NHBs were more likely to present with metastatic cancer in all sites, except for bone, rhabdomyosarcoma, and stomach. NHAPIs were more likely to present with metastatic colorectal, kidney, lung, melanoma and less likely to present with metastatic ovarian cancer. Hispanic AYAs were more likely than non-Hispanics to present with metastatic colorectal, kidney, lung, melanoma, ovarian, and stomach cancer, and soft tissue sarcomas.

#### 3.2.4. Socioeconomic Status

**Figure 6:**
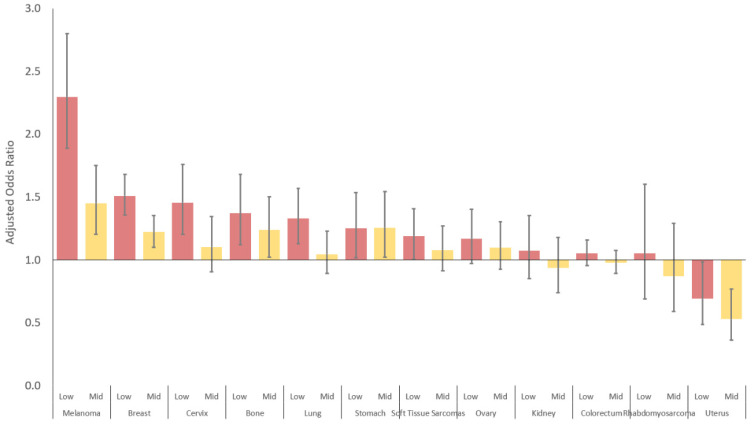
Risk of metastatic disease for low SES AYAs by cancer site. Reference group: high SES.

After adjusting for other sociodemographic variables, low SES AYAs are more likely to present with metastatic melanoma, bone tumors, soft tissue sarcomas, breast, cervical, lung, stomach carcinomas (Figure 6, Table 2). This was most notable for AYAs with melanoma, where the risk of presenting with metastases was 2.3 times higher for low than high SES. There was no SES-related risk for AYAs presenting with metastatic rhabdomyosarcoma or carcinomas of the ovary, kidney, colorectum, and uterus.

## 4. Discussion

Identifying risk factors for presenting with metastatic disease represents a viable strategy for ultimately improving survival among AYAs diagnosed with any of the poor-prognosis cancers included in this “landscape” study. Most importantly, we identified several subgroups of AYAs at significantly higher risk for presenting with metastatic disease as defined by age, sex, race/ethnicity, and/or SES. Interestingly, we found in this analysis that the majority of AYAs with these cancers do not present with metastatic disease but rather with locoregional disease (the two important exceptions being lung and stomach cancer). This latter, somewhat unexpected finding points to the relative importance of specific sociodemographic risk factors within the AYA population rather than some sort of uniform risk broadly belonging to AYAs as a whole. As such, these results can inform future research focused on understanding the roles of tumor and host biology, built environment, health behaviors, provider education, and screening strategies to reduce these risks. Given the well-documented adverse impact of presenting with metastatic disease for these cancers, such efforts have the potential to improve outcomes.

With the known associations of more aggressive forms of cancer in NHB AYAs, such as triple negative breast cancer, and reports of smaller survival improvements among minority AYAs compared with NHW AYAs, we expected to find minority AYAs to have higher risk of metastases in most cancer sites. We found this to be the case for all sites combined, and that this difference was most pronounced in NHAPIs and NHBs. The increased risk of metastases in NHAPIs largely appears to be driven by metastatic lung cancer, which is consistent with studies describing high rates of EGFR-driven metastatic lung cancer in East Asians [43,44]. Interestingly, NHAPIs were also at three times higher risk of metastatic melanoma than NHWs, a finding not well described in the literature [52]. For NHBs, the increased risk of metastases spanned most sites, a pattern not evident in other minority groups. This raises important questions as to the mechanisms driving this recurrent pattern. Potential explanations may involve certain risk alleles identified through genetic ancestry or possibly increased allostatic load as a result of toxic stress secondary to factors such as structural discrimination [53]. Notably, even after adjusting for sex, SES, and age, NHBs carry the highest risk of metastatic melanoma and kidney cancer, two cancer types featuring prominent immunological mechanisms [54,55]. Future studies exploring the impact of toxic stress on the tumor microenvironment and immunologic responses could prove useful for understanding how the macroenvironment may influence the underlying biology driving aggressive, metastatic disease.

Given the importance of fragmented insurance [30] and low patient and provider awareness of AYA cancer in contributing to delayed diagnosis, we expected to see a general pattern of low SES AYAs having a higher risk of metastatic disease than high SES. This was indeed true for all cancers in aggregate and in seven of the 12 cancer sites. However, it appears the overall increased risk was largely driven by metastatic melanoma, where low SES AYAs were over two times more likely to present with metastases than high SES AYAs. This is particularly concerning as the overall incidence of melanoma is greater among high SES populations, but survival is poorer among low SES patients [56]. This suggests a need for stronger prevention efforts in lower SES communities and further exploration of potential delays in diagnosis for this population. In addition to studying potential delays in diagnosis in cancers where low SES is independently associated with metastatic presentation, future studies exploring the mechanisms by which specific factors linked to low SES, such as neighborhood disadvantage and poverty, may contribute to more aggressive forms of these cancers. This link has been explored in literature surrounding impact of low SES and neighborhood depravity in breast cancer outcomes but has not yet been extended to other cancer sites [57]. Notably, low SES did not confer an increased risk of metastases in several cancer sites including ovary, kidney, colorectum, uterus and rhabdomyosarcoma. This would suggest that for these cancers, aggressive underlying biological mechanisms may outweigh the effects of social risk factors.

Given existing literature documenting an increased risk of metastatic disease for men compared to women, we expected men to carry uniformly higher risk of metastatic disease in most cancer sites [41]. This observation was supported when we looked at cancer sites in aggregate and remained true for six out of eight cancer sites that affect both sexes. This suggests an interplay between social and biological risk factors contributing to increased risks of metastatic disease in most cancer sites. Lower health awareness, less health care utilization, and fewer preventive health behaviors in males might lead to diagnostic delays [58]. Additionally, several studies propose various biological mechanisms by which sex impacts risk of metastatic disease including tumor suppressor genes that escape from X-inactivation [59,60,61]. Notably, men with stomach and colorectal cancer were not at higher risk of metastatic disease compared to females, perhaps highlighting unique features of these gastrointestinal cancers related to the underlying biological drivers of metastases or challenges in early diagnosis that spans both sexes.

This study has both strengths and limitations. A key strength is the use of SEER registry data, a robust and reliable resource [62] offering large sample sizes, that permits identification of broad trends across a variety of cancers. This is especially valuable for rarer tumors such as rhabdomyosarcoma, a biologically distinct form of sarcoma prone to present with metastases in AYAs [45]. Indeed, even in this national sample, the number of patients with rhabdomyosarcoma was relatively small, which highlights both the need for and challenges facing large-scale collaborations investigating rare tumors in AYAs. Additionally, although the number of AYAs with metastatic disease of certain racial/ethnic subgroups was limited (e.g., NHBs with melanoma), the magnitude of risk was significant and highlights extremely vulnerable populations passed over by typical prevention efforts. Finally, using a “landscape” approach allows incidence patterns to emerge across cancer types relevant to AYAs as a whole and within subsets, which may be masked in studies focused on single cancers. Potential limitations are those inherent to registry-based research, including possible misclassification of race/ethnicity provided by the reporting site and use of area-based SES rather than individual level. Additionally, the SEER registry currently does not report molecular subtypes of cancer or detailed patient-level treatment and clinical data, which limits study of biologically focused characteristics [63]. Furthermore, an in-depth histological evaluation was not performed as this was outside the scope of the “landscape” approach needed for this exploratory study. In-depth studies evaluating histology and other biological features in a tumor-specific manner are needed.

## 5. Conclusions

Nonetheless, several important implications follow from our results. First, as a whole, AYAs are more likely to present with loco-regional disease than metastatic disease except stomach and lung cancer. While racial/ethnic minorities, lower SES patients and males are generally at higher risk of presenting with metastases, this risk is not uniformly distributed across cancer sites indicating a need for focused efforts to better delineate the mechanisms by which these subsets experience increased risk of metastases. Ongoing work examining the impact of genetic ancestry and intergenerational transmission of cancer predisposition alleles may elucidate the mechanisms by which NHBs carry increased risk across multiple cancer sites. Additionally, future studies exploring the mechanisms by which environmental stressors and social risk factors impact the tumor microenvironment may shed light on the associations of low SES and metastatic disease.

This broad analysis may also provide additional insights regarding AYAs at risk for developing metastatic cancers that are amenable to screening. If there is an element of metastatic disease development driven by delays in diagnosis that could potentially be avoided through early detection, then identification of AYA subsets at highest risk offers an opportunity to focus and enhance targeted screening and education efforts. The risk of metastatic melanoma was greatest in low SES, NHB and male AYAs, highlighting a significant need for improved screening efforts, as well as provider and community education on atypical signs of melanoma in these populations. Within breast cancer, young NHB and low SES women are at highest risk of metastatic disease presentation. While some of this may be driven by aggressive tumor biology with a propensity for early dissemination, efforts may be warranted to improve patient and provider awareness to avoid dismissal of symptoms and facilitation of prompt referrals. Similarly, the increased risk of metastatic cervical cancer in NHB and low SES AYAs may reflect a need for enhanced screening efforts in these vulnerable populations. Interestingly, metastatic colorectal cancer demonstrates similar increased risk across minorities and low and middle SES, perhaps reflecting broader, more uniform issues relating to biological drivers of metastases in AYAs as well as a need for a higher index of suspicion among clinicians. Future cancer-specific studies focused on exploring the connection between sociodemographic risk factors and biological drivers of metastases have the potential to further inform public health and screening efforts to facilitate early detection of these deadly diseases.

## Figures and Tables

**Table 1:** Proportion of Metastatic Disease by Sociodemographic Subgroup: Surveillance, Epidemiology, and End Results Program (2000–2016).

Cancer Site	Sociodemographic Variable	Subgroup	Locoregional (N, %)	Metastatic (N, %)	Chi-Square *p*-Value
All Cancer Sites Combined	Age *	15–29 years	28,755 (87)	4324 (13)	0.001
		30–39 years	91,072 (88)	12,869 (12)	
	Sex *	Male	28,114 (81)	6618 (19)	0.000
		Female	91,713 (90)	10,575 (10)	
	Race/Ethnicity *	NHW	74,297 (90)	8471 (10)	0.000
		NHB	12,152 (82)	2743 (18)	
		Hispanic	20,969 (84)	4018 (16)	
		NHAPI	8965 (83)	1807 (17)	
	SES *	High	41,996 (90)	4765 (10)	0.000
		Middle	38,133 (88)	5307 (12)	
		Low	31,669 (84)	5951 (16)	
All Cancer Sites Combined: Exclude Female Only Sites	Age *	15–29 years	20,804 (86)	3416 (14)	0.000
		30–39 years	40,872 (83)	8539 (17)	
	Sex *	Male	28,114 (81)	6618 (19)	0.000
		Female	33,562 (86)	5337 (14)	
	Race/Ethnicity *	NHW	42,956 (88)	5942 (12)	0.000
		NHB	4618 (73)	1732 (27)	
		Hispanic	8586 (75)	2847 (25)	
		NHAPI	2911 (69)	1327 (31)	
	SES *	High	22,916 (87)	3350 (13)	0.000
		Middle	19,706 (84)	3678 (16)	
		Low	14,711 (78)	4057 (22)	
Bone	Age *	15–29 years	2282 (76)	719 (24)	0.000
		30–39 years	987 (83)	200 (17)	
	Sex *	Male	1893 (76)	607 (24)	0.000
		Female	1376 (82)	312 (18)	
	Race/Ethnicity	NHW	1819 (79)	484 (21)	0.081
		NHB	361 (79)	97 (21)	
		Hispanic	780 (75)	259 (25)	
		NHAPI	258 (79)	70 (21)	
	SES *	High	1053 (81)	248 (19)	0.003
		Middle	1057 (78)	305 (22)	
		Low	938 (75)	306 (25)	
Breast	Age *	15–29 years	3784 (91)	369 (9)	0.000
		30–39 years	34,150 (94)	2303 (6)	
	Race/Ethnicity *	NHW	20,786 (94)	1224 (6)	0.000
		NHB	5453 (89)	648 (11)	
		Hispanic	7014 (93)	522 (7)	
		NHAPI	4256 (94)	253 (6)	
	SES *	High	14,216 (95)	770 (5)	0.000
		Middle	11,902 (93)	840 (7)	
		Low	9603 (91)	926 (9)	
Cervix	Age *	15–29 years	2816 (94)	172 (6)	0.013
		30–39 years	10,138 (93)	769 (7)	
	Race/Ethnicity *	NHW	6999 (94)	471 (6)	0.000
		NHB	1421 (90)	158 (10)	
		Hispanic	3472 (94)	240 (6)	
		NHAPI	809 (93)	61 (7)	
	SES *	High	4774 (92)	427 (8)	0.000
		Middle	4057 (94)	266 (6)	
		Low	3141 (95)	181 (5)	
Colorectum	Age *	15–29 years	2743 (78)	770 (22)	0.000
		30–39 years	8997 (74)	3147 (26)	
	Sex	Male	6108 (75)	2002 (25)	0.320
		Female	5632 (75)	1915 (25)	
	Race/Ethnicity *	NHW	6666 (76)	2098 (24)	0.000
		NHB	1523 (73)	555 (27)	
		Hispanic	2243 (73)	825 (27)	
		NHAPI	1059 (72)	402 (28)	
	SES	High	3672 (75)	1197 (25)	0.081
		Middle	3749 (76)	1215 (24)	
		Low	3510 (74)	1249 (26)	
Kidney	Age *	15–29 years	1102 (87)	161 (13)	0.000
		30–39 years	5461 (93)	441 (7)	
	Sex *	Male	3754 (91)	390 (9)	0.000
		Female	2809 (93)	212 (7)	
	Race/Ethnicity *	NHW	3752 (94)	241 (6)	0.000
		NHB	790 (81)	185 (19)	
		Hispanic	1486 (92)	124 (8)	
		NHAPI	387 (90)	45 (10)	
	SES *	High	1893 (93)	153 (7)	0.001
		Middle	2155 (93)	171 (7)	
		Low	2057 (90)	228 (10)	
Lung	Age *	15–29 years	528 (61)	332 (39)	0.000
		30–39 years	1357 (37)	2313 (63)	
	Sex *	Male	839 (38)	1379 (62)	0.000
		Female	1046 (45)	1266 (55)	
	Race/Ethnicity *	NHW	1215 (46)	1429 (54)	0.000
		NHB	234 (38)	375 (62)	
		Hispanic	290 (41)	415 (59)	
		NHAPI	126 (24)	408 (76)	
	SES *	High	578 (44)	734 (56)	0.001
		Middle	604 (43)	796 (57)	
		Low	546 (37)	912 (63)	
Melanoma	Age *	15–29 years	10,259 (98)	229 (2)	0.040
		30–39 years	18,625 (97)	491 (3)	
	Sex *	Male	10,866 (96)	467 (4)	0.000
		Female	18,018 (99)	253 (1)	
	Race/Ethnicity *	NHW	25,098 (98)	598 (2)	0.000
		NHB	143 (89)	17 (11)	
		Hispanic	1490 (95)	86 (5)	
		NHAPI	248 (94)	16 (6)	
	SES *	High	12,942 (98)	221 (2)	0.000
		Middle	9207 (98)	235 (2)	
		Low	4701 (96)	205 (4)	
Ovary	Age *	15–29 years	686 (67)	334 (33)	0.000
		30–39 years	1527 (59)	1075 (41)	
	Race/Ethnicity *	NHW	1237 (63)	742 (37)	0.000
		NHB	158 (48)	173 (52)	
		Hispanic	466 (57)	353 (43)	
		NHAPI	328 (71)	132 (29)	
	SES *	High	734 (65)	403 (35)	0.001
		Middle	751 (62)	468 (38)	
		Low	599 (57)	453 (43)	
RMS	Age	15–29 years	300 (55)	247 (45)	0.827
		30–39 years	81 (56)	64 (44)	
	Sex *	Male	206 (50)	206 (50)	0.001
		Female	175 (63)	105 (38)	
	Race/Ethnicity	NHW	193 (55)	155 (45)	0.357
		NHB	71 (59)	50 (41)	
		Hispanic	90 (54)	77 (46)	
		NHAPI	20 (43)	26 (57)	
	SES	High	117 (52)	106 (48)	0.583
		Middle	120 (57)	89 (43)	
		Low	112 (54)	94 (46)	
Soft Tissue Sarcomas	Age *	15–29 years	3388 (85)	596 (15)	0.000
		30–39 years	4338 (88)	598 (12)	
	Sex *	Male	3808 (84)	699 (16)	0.000
		Female	3918 (89)	495 (11)	
	Race/Ethnicity *	NHW	3833 (88)	511 (12)	0.000
		NHB	1323 (84)	251 (16)	
		Hispanic	1738 (84)	322 (16)	
		NHAPI	625 (86)	100 (14)	
	SES *	High	2315 (88)	306 (12)	0.000
		Middle	2461 (87)	369 (13)	
		Low	2408 (85)	433 (15)	
Stomach	Age *	15–29 years	202 (36)	362 (64)	0.000
		30–39 years	1026 (44)	1285 (56)	
	Sex	Male	640 (42)	868 (58)	0.756
		Female	588 (43)	779 (57)	
	Race/Ethnicity *	NHW	380 (47)	426 (53)	0.001
		NHB	173 (46)	202 (54)	
		Hispanic	469 (39)	739 (61)	
		NHAPI	188 (42)	260 (58)	
	SES *	High	346 (47)	385 (53)	0.018
		Middle	353 (41)	498 (59)	
		Low	439 (41)	630 (59)	
Uterus	Age	15–29 years	665 (95)	33 (5)	0.371
		30–39 years	4385 (96)	183 (4)	
	Race/Ethnicity	NHW	2319 (96)	92 (4)	0.082
		NHB	502 (94)	32 (6)	
		Hispanic	1431 (96)	56 (4)	
		NHAPI	661 (95)	34 (5)	
	SES *	High	989 (94)	61 (6)	0.002
		Middle	1717 (97)	55 (3)	
		Low	1982 (96)	88 (4)	

* Statistically significant *p*-value (*p* < 0.05). The differences in proportions of the subgroups were compared for each sociodemographic variables using chi-square analysis. Abbreviations: NHW = non-Hispanic White; NHB = non-Hispanic Black; NHAPI = non-Hispanic Asian Pacific Islander; RMS = Rhabdomyosarcoma; SES = socioeconomic status.

**Table 2:** Crude and Adjusted Odds Ratio for Metastatic Disease Presentation: Surveillance, Epidemiology, and End Results Program (2000–2016).

Cancer Site	Sociodemographic Category	Subgroup	Crude Odds Ratio (95% CI)	Adjusted Odds Ratio (95% CI)
All sites combined	Age	30–39 years	--	--
		15–29 years	**1.07 (1.03–1.11)**	1.00 (0.96–1.04)
	Sex	Female	--	--
		Male	**2.05 (1.98–2.12)**	**2.18 (2.11–2.26)**
	Race/Ethnicity	NHW	--	--
		NHB	**1.95 (1.86–2.04)**	**1.86 (1.77–1.96)**
		Hispanic	**1.66 (1.59–1.72)**	**1.61 (1.54–1.68)**
		NHAPI	**1.75 (1.66–1.85)**	**1.93 (1.82–2.04)**
	SES	High	--	--
		Middle	**1.22 (1.17–1.27)**	**1.16 (1.11–1.21)**
		Low	**1.63 (1.57–1.70)**	**1.42 (1.36–1.48)**
Bone	Age	30–39 years	--	--
		15–29 years	**1.55 (1.31–1.85)**	**1.51 (1.26–1.82)**
	Sex	Female	--	--
		Male	**1.41 (1.21–1.65)**	**1.34 (1.14–1.57)**
	Race/Ethnicity	NHW	--	--
		NHB	1.01 (0.79–1.29)	0.91 (0.70–1.19)
		Hispanic	**1.25 (1.05–1.48)**	1.12 (0.93–1.35)
		NHAPI	1.02 (0.77–1.35)	1.08 (0.81–1.45)
	SES	High	--	--
		Middle	**1.23 (1.02–1.48)**	**1.24 (1.02–1.50)**
		Low	**1.39 (1.15–1.67)**	**1.37 (1.12–1.68)**
Breast	Age	30–39 years	--	--
		15–29 years	**1.43 (1.28–1.61)**	**1.36 (1.20–1.53)**
	Race/Ethnicity	NHW	--	--
		NHB	**2.01 (1.82–2.22)**	**1.72 (1.55–1.92)**
		Hispanic	**1.26 (1.13–1.40)**	**1.12 (1.00–1.25)**
		NHAPI	1.01 (0.88–1.16)	1.04 (0.90–1.20)
	SES	High	--	--
		Middle	**1.30 (1.17–1.43)**	**1.22 (1.10–1.35)**
		Low	**1.77 (1.60–1.95)**	**1.51 (1.36–1.68)**
Cervix	Age	30–39 years	--	--
		15–29 years	**0.79 (0.67–0.94)**	**0.82 (0.68–0.98)**
	Race/Ethnicity	NHW	--	--
		NHB	**1.62 (1.34–1.95)**	**1.50 (1.22–1.83)**
		Hispanic	1.02 (0.87–1.20)	1.02 (0.86–1.21)
		NHAPI	1.14 (0.86–1.50)	1.18 (0.89–1.57)
	SES	High	--	--
		Middle	1.13 (0.93–1.37)	1.10 (0.90–1.34)
		Low	**1.53 (1.28–1.83)**	**1.46 (1.20–1.76)**
Colorectum	Age	30–39 years	--	--
		15–29 years	**0.80 (0.73–0.88)**	**0.79 (0.72–0.87)**
	Sex	Female	--	--
		Male	0.96 (0.90–1.03)	0.97 (0.90–1.04)
	Race/Ethnicity	NHW	--	--
		NHB	**1.14 (1.02–1.27)**	**1.16 (1.04–1.31)**
		Hispanic	**1.16 (1.06–1.27)**	**1.16 (1.05–1.28)**
		NHAPI	**1.20 (1.06–1.36)**	**1.22 (1.07–1.38)**
	SES	High	--	--
		Middle	0.99 (0.90–1.09)	0.98 (0.89–1.07)
		Low	1.08 (0.98–1.18)	1.05 (0.95–1.16)
Kidney	Age	30–39 years	--	--
		15–29 years	**1.78 (1.47–2.16)**	**1.81 (1.47–2.22)**
	Sex	Female	--	--
		Male	**1.37 (1.15–1.63)**	**1.45 (1.20–1.75)**
	Race/Ethnicity	NHW	--	--
		NHB	**3.57 (2.91–4.39)**	**3.61 (2.88–4.53)**
		Hispanic	**1.29 (1.03–1.62)**	**1.33 (1.05–1.69)**
		NHAPI	**1.81 (1.29–2.52)**	**2.02 (1.43–2.84)**
	SES	High	--	--
		Middle	0.98 (0.78–1.23)	0.93 (0.74–1.18)
		Low	**1.36 (1.10–1.69)**	**1.07 (0.85–1.35)**
Lung	Age	30–39 years	--	--
		15–29 years	**0.37 (0.32–0.43)**	**0.36 (0.31–0.43)**
	Sex	Female	--	--
		Male	**1.36 (1.21–1.53)**	**1.35 (1.19–1.53)**
	Race/Ethnicity	NHW	--	--
		NHB	**1.36 (1.14–1.63)**	**1.29 (1.06–1.58)**
		Hispanic	**1.22 (1.03–1.44)**	**1.32 (1.10–1.59)**
		NHAPI	**2.75 (2.22–3.41)**	**2.97 (2.36–3.73)**
	SES	High	--	--
		Middle	1.04 (0.89–1.21)	1.05 (0.89–1.23)
		Low	**1.32 (1.13–1.53)**	**1.33 (1.13–1.57)**
Melanoma	Age	30–39 years	--	--
		15–29 years	**0.85 (0.72–0.99)**	**0.89 (0.75–1.05)**
	Sex	Female	--	--
		Male	**3.06 (2.62–3.57)**	**3.11 (2.64–3.66)**
	Race/Ethnicity	NHW	--	--
		NHB	**4.99 (3.00–8.30)**	**4.04 (2.32–7.04)**
		Hispanic	**2.42 (1.92–3.05)**	**2.37 (1.85–3.04)**
		NHAPI	**2.71 (1.62–4.52)**	**2.99 (1.75–5.12)**
	SES	High	--	--
		Middle	**1.49 (1.24–1.80)**	**1.45 (1.20–1.75)**
		Low	**2.55 (2.11–3.10)**	**2.30 (1.89–2.80)**
Ovary	Age	30–39 years	--	--
		15–29 years	**0.70 (0.60–0.81)**	**0.70 (0.60–0.83)**
	Race/Ethnicity	NHW	--	--
		NHB	**1.71 (1.36–2.14)**	**1.79 (1.40–2.30)**
		Hispanic	**1.27 (1.08–1.50)**	**1.28 (1.07–1.52)**
		NHAPI	**0.69 (0.56–0.86)**	**0.66 (0.53–0.83)**
	SES	High	--	--
		Middle	1.11 (0.94–1.30)	1.10 (0.92–1.30)
		Low	**1.33 (1.12–1.58)**	**1.17 (0.97–1.40)**
Rhabdomyosarcoma	Age	30–39 years	--	--
		15–29 years	1.04 (0.72–1.51)	1.13 (0.77–1.68)
	Sex	Female	--	--
		Male	**1.67 (1.22–2.27)**	**1.75 (1.27–2.43)**
	Race/Ethnicity	NHW	--	--
		NHB	0.88 (0.58–1.33)	0.81 (0.51–1.31)
		Hispanic	1.07 (0.74–1.54)	1.10 (0.74–1.65)
		NHAPI	1.62 (0.87–3.01)	1.70 (0.89–3.26)
	SES	High	--	--
		Middle	0.82 (0.56–1.20)	0.87 (0.59–1.29)
		Low	0.93 (0.63–1.35)	1.05 (0.69–1.60)
Soft Tissue Sarcomas	Age	30–39 years	--	--
		15–29 years	**1.28 (1.13–1.44)**	**1.26 (1.11–1.44)**
	Sex	Female	--	--
		Male	**1.45 (1.28–1.64)**	**1.47 (1.29–1.67)**
	Race/Ethnicity	NHW	--	--
		NHB	**1.42 (1.21–1.68)**	**1.40 (1.17–1.67)**
		Hispanic	**1.39 (1.20–1.62)**	**1.35 (1.15–1.58)**
		NHAPI	1.20 (0.95–1.51)	1.20 (0.95–1.53)
	SES	High	--	--
		Middle	1.13 (0.96–1.33)	1.08 (0.91–1.27)
		Low	**1.36 (1.16–1.59)**	**1.19 (1.01–1.41)**
Stomach	Age	30–39 years	--	--
		15–29 years	**1.34 (1.12–1.60)**	**1.43 (1.17–1.75)**
	Sex	Female	--	--
		Male	1.02 (0.88–1.17)	1.05 (0.90–1.23)
	Race/Ethnicity	NHW	--	--
		NHB	1.10 (0.87–1.40)	0.92 (0.71–1.19)
		Hispanic	**1.37 (1.15–1.63)**	**1.28 (1.05–1.56)**
		NHAPI	**1.27 (1.02–1.59)**	**1.19 (0.93–1.52)**
	SES	High	--	--
		Middle	**1.25 (1.03–1.51)**	**1.26 (1.02–1.54)**
		Low	**1.25 (1.05–1.51)**	**1.25 (1.02–1.53)**
Uterus	Age	30–39 years	--	--
		15–29 years	1.11 (0.76–1.62)	1.27 (0.86–1.88)
	Race/Ethnicity	NHW	--	--
		NHB	**1.58 (1.04–2.38)**	**1.59 (1.03–2.46)**
		Hispanic	0.96 (0.69–1.35)	0.98 (0.69–1.40)
		NHAPI	1.28 (0.85–1.91)	1.18 (0.78–1.79)
	SES	High	--	--
		Middle	**0.52 (0.36–0.75)**	**0.53 (0.36–0.77)**
		Low	**0.72 (0.52–1.01)**	**0.69 (0.49–0.98)**

Bolded cells denote a statistically significant *p*-value (*p* < 0.05). Abbreviations: NHW = non-Hispanic White; NHB = non-Hispanic Black; NHAPI = non-Hispanic Asian Pacific Islander; SES = socioeconomic status.

## Data Availability

Data was accessed from the SEER Census-Tract Level SES and Rurality Database and is publicly available upon request at https://seer.cancer.gov/seertrack/data/request/ (accessed on 22 September 2022).

## References

[1] Moke D.J., Tsai K., Hamilton A.S., Hwang A., Liu L., Freyer D.R., Deapen D. (2019). Emerging Cancer Survival Trends, Disparities, and Priorities in Adolescents and Young Adults: A California Cancer Registry-Based Study. JNCI Cancer Spectr..

[2] Miller K.D., Fidler-Benaoudia M., Keegan T.H., Hipp H.S., Jemal A., Siegel R.L. (2020). Cancer statistics for adolescents and young adults, 2020. CA A Cancer J. Clin..

[3] Murphy C.C., Lupo P.J., Roth M.E., Winick N.J., Pruitt S.L. (2021). Disparities in cancer survival among adolescents and young adults: A population-based study of 88,000 patients. JNCI J. Natl. Cancer Inst..

[4] Ellis L., Canchola A.J., Spiegel D., Ladabaum U., Haile R., Gomez S.L. (2018). Racial and Ethnic Disparities in Cancer Survival: The Contribution of Tumor, Sociodemographic, Institutional, and Neighborhood Characteristics. J. Clin. Oncol..

[5] Liu L.H.A., Moke D., Tsai K.Y., Wojcik K.Y., Cockburn M., Deapen D. (2017). Cancer in Los Angeles County: Survival among Adolescents and Young Adults 1988–2014.

[6] Keegan T.H., Ries L.A., Barr R.D., Geiger A.M., Dahlke D.V., Pollock B.H., Bleyer W.A., for the National Cancer Institute Next Steps for Adolescent and Young Adult Oncology Epidemiology Working Group (2016). Comparison of cancer survival trends in the United States of adolescents and young adults with those in children and older adults. Cancer.

[7] Tricoli J.V., Bleyer A. (2018). Adolescent and Young Adult Cancer Biology. Cancer J..

[8] Tricoli J.V., Seibel N.L., Blair D.G., Albritton K., Hayes-Lattin B. (2011). Unique Characteristics of Adolescent and Young Adult Acute Lymphoblastic Leukemia, Breast Cancer, and Colon Cancer. JNCI J. Natl. Cancer Inst..

[9] Bleyer A., Barr R., Hayes-Lattin B., Thomas D., Ellis C., Anderson B., on behalf of the Biology and Clinical Trials Subgroups of the US National Cancer Institute Progress Review Group in Adolescent and Young Adult Oncology (2008). The distinctive biology of cancer in adolescents and young adults. Nat. Rev. Cancer.

[10] Martin S., Ulrich C., Munsell M., Taylor S., Lange G., Bleyer A. (2007). Delays in Cancer Diagnosis in Underinsured Young Adults and Older Adolescents. Oncologist.

[11] Fardell J.E., Patterson P., Wakefield C.E., Signorelli C., Cohn R., Anazodo A., Zebrack B., Sansom-Daly U. (2018). A Narrative Review of Models of Care for Adolescents and Young Adults with Cancer: Barriers and Recommendations. J. Adolesc. Young Adult Oncol..

[12] Leiner J., Le Loarer F. (2020). The current landscape of rhabdomyosarcomas: An update. Virchows Arch..

[13] Newman L.A., Jenkins B., Chen Y., Oppong J.K., Adjei E., Jibril A.S., Hoda S., Cheng E., Chitale D., Bensenhaver J.M. (2019). Hereditary Susceptibility for Triple-Negative Breast Cancer Associated with Western Sub-Saharan African Ancestry. Ann. Surg..

[14] Martini R., Newman L., Davis M. (2021). Breast cancer disparities in outcomes; unmasking biological determinants associated with racial and genetic diversity. Clin. Exp. Metastasis.

[15] Obeng-Gyasi S., Tarver W., Carlos R.C., Andersen B.L. (2021). Allostatic load: A framework to understand breast cancer outcomes in Black women. NPJ Breast Cancer.

[16] Hendifar A., Yang D., Lenz F., Lurje G., Pohl A., Lenz C., Ning Y., Zhang W., Lenz H.-J. (2009). Gender Disparities in Metastatic Colorectal Cancer Survival. Clin. Cancer Res..

[17] White A., Ironmonger L., Steele R.J.C., Ormiston-Smith N., Crawford C., Seims A. (2018). A review of sex-related differences in colorectal cancer incidence, screening uptake, routes to diagnosis, cancer stage and survival in the UK. BMC Cancer.

[18] Hunt J.D., van der Hel O.L., McMillan G.P., Boffetta P., Brennan P. (2005). Renal cell carcinoma in relation to cigarette smoking: Meta-analysis of 24 studies. Int. J. Cancer.

[19] Brennan P., Van Der Hel O., Moore L.E., Zaridze D., Matveev V., Holcatova I., Janout V., Kollarova H., Foretova L., Szeszenia-Dabrowska N. (2008). Tobacco smoking, body mass index, hypertension, and kidney cancer risk in central and eastern Europe. Br. J. Cancer.

[20] Viñal D., Martínez D., Higuera O., De Castro J. (2021). Genomic profiling in non-small-cell lung cancer in young patients. A Syst. Rev. ESMO Open.

[21] Kreuzer M., Kreienbrock L., Gerken M., Heinrich J., Bruske-Hohlfeld I., Muller K.-M., Wichmann H.E. (1998). Risk Factors for Lung Cancer in Young Adults. Am. J. Epidemiol..

[22] Petrick J.L., Jensen B.W., Sørensen T.I., Cook M.B., Baker J.L. (2019). Overweight Patterns Between Childhood and Early Adulthood and Esophageal and Gastric Cardia Adenocarcinoma Risk. Obesity.

[23] Garrett B.E., Martell B.N., Caraballo R.S., King B.A. (2019). Socioeconomic Differences in Cigarette Smoking Among Sociodemographic Groups. Prev. Chronic Dis..

[24] Pavela G., Lewis D.W., Locher J., Allison D.B. (2016). Socioeconomic Status, Risk of Obesity, and the Importance of Albert J. Stunkard. Curr. Obes. Rep..

[25] Malaty H.M., Graham D.Y. (1994). Importance of childhood socioeconomic status on the current prevalence of Helicobacter pylori infection. Gut.

[26] Clifford J.S., Lu J., Blondino C.T., Do E.K., Prom-Wormley E.C. (2022). The Association Between Health Literacy and Tobacco Use: Results from a Nationally Representative Survey. J. Community Health.

[27] Lachowycz K., Jones A.P. (2011). Greenspace and obesity: A systematic review of the evidence. Obes. Rev..

[28] Cooksey-Stowers K., Schwartz M., Brownell K. (2017). Food Swamps Predict Obesity Rates Better Than Food Deserts in the United States. Int. J. Environ. Res. Public Health.

[29] Mou J., Bolieu E.L., Pflugeisen B.M., Amoroso P.J., Devine B., Baldwin L.M., Frank L.L., Johnson R.H. (2019). Delay in Treatment After Cancer Diagnosis in Adolescents and Young Adults: Does Facility Transfer Matter?. J. Adolesc. Young Adult Oncol..

[30] Keegan T.H., Parsons H.M., Chen Y., Maguire F.B., Morris C.R., Parikh-Patel A., Kizer K.W., Wun T. (2019). Impact of Health Insurance on Stage at Cancer Diagnosis Among Adolescents and Young Adults. JNCI J. Natl. Cancer Inst..

[31] Herbert A., Lyratzopoulos G., Whelan J., Taylor R.M., Barber J., Gibson F., Fern L.A. (2018). Diagnostic timeliness in adolescents and young adults with cancer: A cross-sectional analysis of the BRIGHTLIGHT cohort. Lancet Child Adolesc. Health.

[32] McDaniel C.C., Hallam H.H., Cadwallader T., Lee H.Y., Chou C. (2021). Persistent racial disparities in cervical cancer screening with Pap test. Prev. Med. Rep..

[33] USPSTF (2022). Skin Cancer Screening Guidelines. https://www.uspreventiveservicestaskforce.org/uspstf/recommendation/skin-cancer-screening#:~:text=The%20USPSTF%20recommends%20that%20children.

[34] Tao L., Gomez S.L., Keegan T.H., Kurian A.W., Clarke C.A. (2015). Breast Cancer Mortality in African-American and Non-Hispanic White Women by Molecular Subtype and Stage at Diagnosis: A Population-Based Study. Cancer Epidemiol. Biomark. Prev..

[35] Sineshaw H.M., Gaudet M., Ward E.M., Flanders W.D., Desantis C., Lin C.C., Jemal A. (2014). Association of race/ethnicity, socioeconomic status, and breast cancer subtypes in the National Cancer Data Base (2010–2011). Breast Cancer Res. Treat..

[36] Keegan T.H.M., Press D.J., Tao L., DeRouen M.C., Kurian A.W., Clarke C.A., Gomez S.L. (2013). Impact of breast cancer subtypes on 3-year survival among adolescent and young adult women. Breast Cancer Res..

[37] Press D.J., Miller M.E., Liederbach E., Yao K., Huo D. (2017). De novo metastasis in breast cancer: Occurrence and overall survival stratified by molecular subtype. Clin. Exp. Metastasis.

[38] Murphy C.C., Wallace K., Sandler R.S., Baron J.A. (2019). Racial Disparities in Incidence of Young-Onset Colorectal Cancer and Patient Survival. Gastroenterology.

[39] Stewart S.L., Wike J.M., Kato I., Lewis D.R., Michaud F. (2006). A population-based study of colorectal cancer histology in the United States, 1998–2001. Cancer.

[40] Holowatyj A.N., Lewis M.A., Pannier S.T., Kirchhoff A.C., Hardikar S., Figueiredo J.C., Huang L.C., Shibata D., Schmit S.L., Ulrich C.M. (2019). Clinicopathologic and Racial/Ethnic Differences of Colorectal Cancer Among Adolescents and Young Adults. Clin. Transl. Gastroenterol..

[41] Wang R., Wang M.J., Ping J. (2015). Clinicopathological Features and Survival Outcomes of Colorectal Cancer in Young Versus Elderly: A Population-Based Cohort Study of SEER 9 Registries Data (1988–2011). Medicine.

[42] O’Connell J.B., Maggard M.A., Livingston E.H., Yo C.K. (2004). Colorectal cancer in the young. Am. J. Surg..

[43] Shi Y., Au J.S.-K., Thongprasert S., Srinivasan S., Tsai C.-M., Khoa M.T., Heeroma K., Itoh Y., Cornelio G., Yang P.-C. (2014). A Prospective, Molecular Epidemiology Study of EGFR Mutations in Asian Patients with Advanced Non–Small-Cell Lung Cancer of Adenocarcinoma Histology (PIONEER). J. Thorac. Oncol..

[44] Zhou F., Zhou C. (2018). Lung cancer in never smokers—The East Asian experience. Transl. Lung Cancer Res..

[45] Bhutada J.S., Hwang A., Liu L., Deapen D., Freyer D.R. (2021). Poor-Prognosis Metastatic Cancers in Adolescents and Young Adults: Incidence Patterns, Trends, and Disparities. JNCI Cancer Spectr..

[46] SEER Summary Staging Definition. https://training.seer.cancer.gov/staging/systems/summary/distant.html#:~:text=Definition%3A,%2C%20disseminated%2C%20diffuse%2C%20metastatic.

[47] Skapek S.X., Ferrari A., Gupta A.A., Lupo P.J., Butler E., Shipley J., Barr F.G., Hawkins D.S. (2019). Rhabdomyosarcoma. Nat. Rev. Dis. Primers.

[48] Shiels M.S., Cole S.R., Wegner S., Armenian H., Chmiel J.S., Ganesan A., Marconi V.C., Martinez-Maza O., Martinson J., Weintrob A. (2008). Effect of HAART on Incident Cancer and Noncancer AIDS Events Among Male HIV Seroconverters. J. Acquir. Immune Defic. Syndr..

[49] Liu L., Deapen D., Bernstein L. (1998). Socioeconomic status and cancers of the female breast and reproductive organs: A comparison across racial/ethnic populations in Los Angeles County, California (United States). Cancer Causes Control.

[50] Yost K., Perkins C., Cohen R., Morris C., Wright W. (2001). Socioeconomic status and breast cancer incidence in California for different race/ethnic groups. Cancer Causes Control.

[51] SEER*Stat Surveillance Research Program, National Cancer Institute SEER*Stat Software, Version <8.3>. https://seer.cancer.gov/seerstat.

[52] Zheng Y.J., Ho C., Lazar A., Ortiz-Urda S. (2021). Poor melanoma outcomes and survival in Asian American and Pacific Islander patients. J. Am. Acad. Dermatol..

[53] Duru O.K., Harawa N.T., Kermah D., Norris K.C. (2012). Allostatic Load Burden and Racial Disparities in Mortality. J. Natl. Med. Assoc..

[54] Passarelli A., Mannavola F., Stucci L.S., Tucci M., Silvestris F. (2017). Immune system and melanoma biology: A balance between immunosurveillance and immune escape. Oncotarget.

[55] Díaz-Montero C.M., Rini B.I., Finke J.H. (2020). The immunology of renal cell carcinoma. Nat. Rev. Nephrol..

[56] Zell J.A., Cinar P., Mobasher M., Ziogas A., Meyskens F.L., Anton-Culver H. (2008). Survival for Patients with Invasive Cutaneous Melanoma Among Ethnic Groups: The Effects of Socioeconomic Status and Treatment. J. Clin. Oncol..

[57] Saini G., Ogden A., McCullough L.E., Torres M., Rida P., Aneja R. (2019). Disadvantaged neighborhoods and racial disparity in breast cancer outcomes: The biological link. Cancer Causes Control.

[58] Wang Y., Freemantle N., Nazareth I., Hunt K. (2014). Gender Differences in Survival and the Use of Primary Care Prior to Diagnosis of Three Cancers: An Analysis of Routinely Collected UK General Practice Data. PLoS ONE.

[59] Arnold A.P., Disteche C.M. (2018). Sexual Inequality in the Cancer Cell. Cancer Res..

[60] Dunford A., Weinstock D.M., Savova V., Schumacher S.E., Cleary J.P., Yoda A., Sullivan T.J., Hess J.M., Gimelbrant A.A., Beroukhim R. (2017). Tumor-suppressor genes that escape from X-inactivation contribute to cancer sex bias. Nat. Genet..

[61] Klein S.L., Flanagan K.L. (2016). Sex differences in immune responses. Nat. Rev. Immunol..

[62] SEER QI Process. https://seer.cancer.gov/qi/process.html.

[63] Pollock B.H. (2020). What’s Missing in the Assessment of Adolescent and Young Adult (AYA) Cancer Outcomes?. J. Natl. Cancer Inst..

